# Prevalence and Characterization of *Cryptosporidium* Species in Tibetan Antelope (*Pantholops hodgsonii*)

**DOI:** 10.3389/fcimb.2021.713873

**Published:** 2021-09-06

**Authors:** Si-Yuan Qin, He-Ting Sun, Chuang Lyu, Jun-Hui Zhu, Zhen-Jun Wang, Tao Ma, Quan Zhao, Yun-Gang Lan, Wen-Qi He

**Affiliations:** ^1^Key Laboratory of Zoonosis Research, Ministry of Education, College of Veterinary Medicine, Jilin University, Changchun, China; ^2^General Monitoring Station for Wildlife-Borne Infectious Diseases, State Forestry and Grass Administration, Shenyang, China; ^3^Animal Health Center, Shandong New Hope Liuhe Group Co. Ltd., Qingdao, China; ^4^Animal Health Center, Qingdao Jiazhi Biotechnology Co. Ltd., Qingdao, China; ^5^College of Life Science, Changchun Sci-Tech University, Shuangyang, China

**Keywords:** *Cryptosporidium*, Tibetan antelope (*Pantholops hodgsonii*), prevalence, characterization, PCR

## Abstract

*Cryptosporidium* is an enteric *apicomplexan* parasite, which can infect multiple mammals including livestock and wildlife. Tibetan Antelope (*Pantholops hodgsonii*) is one of the most famous wildlife species, that belongs to the first class protected wild animals in China. However, it has not been known whether Tibetan Antelope is infected with *Cryptosporidium* so far. The objective of the present study was to determine the prevalence and characterization of *Cryptosporidium* species infection in Tibetan Antelope and the corresponding species by using molecular biological method. In the current study, a total of 627 fecal samples were randomly collected from Tibetan Antelope in the Tibet Autonomous Region (2019–2020), and were examined by PCR amplification of the small subunit ribosomal RNA (SSU rRNA) gene. Among 627 samples, 19 (3.03%, 19/627) were examined as *Cryptosporidium*-positive, with 7 (2.33%, 7/300) in females and 12 (3.67%, 12/327) in males. The analysis of SSU rRNA gene sequence suggested that only two *Cryptosporidium* species, namely, *C. xiaoi* and *C. ubiquitum*, were identified in this study. This is the first evidence for an existence of *Cryptosporidium* in Tibetan Antelope. These findings extend the host range for *Cryptosporidium* spp. and also provide important data support for prevention and control of *Cryptosporidium* infection in Tibetan Antelope.

## Introduction

*Cryptosporidium*, the causative agent of cryptosporidiosis, causes an intestinal disease in a wide range of hosts worldwide, including wildlife, livestock, and humans. Human infection with *Cryptosporidium* is usually through a close contact with the infected animals or consuming contaminated water or food (Rossignol, 2010). At least 38 species and over 70 genotypes of *Cryptosporidium* can infect humans and animals (Deng et al., 2020). Among them, more than 20 have been considered as zoonotic potential risks, including *C. hominis*, *C. parvum*, *C. meleagridis*, *C. felis*, *C. canis*, *C. cuniculus*, *C. ubiquitum*, *C. viatorum*, *C. muris*, *C. suis*, *C. fayeri*, *C. andersoni*, *C. bovis*, *C. scrofarum*, *C. xiaoi*, *C. tyzzeri*, *C. erinaceid*, and *C. horse*, *C. skunk*, and *C. chipmunk* I genotype (Ren et al., 2012; Adamu et al., 2014; Koehler et al., 2014; Kváč et al., 2014; Ma et al., 2014; Qi et al., 2014; Qin et al., 2014; Yang et al., 2014; Galuppi et al., 2015; Lin et al., 2015; Yan et al., 2017; Firoozi et al., 2019; Takaki et al., 2020; Xu et al., 2020). *C. hominis* and *C. parvum* were most frequently found in human. *C. xiaoi* was generally considered as *C. bovis*-like genotype or *C. bovis* when Fayer and Santín identified it as a new species in 2009 based on morphology and molecular methods (Fayer and Santín, 2009).

Since *C. xiaoi* and *C. ubiquitum* were recognized firstly in sheep, many researches were focused on the prevalence of *C. xiaoi* and *C. ubiquitum* in humans and other animals which have closer relationship with the sheep like bovine and cervine. To date, *C. xiaoi* infection in sheep has been reported in many countries, including Ireland, Kuwait, Australia, Norway, Spain, France, Greece, Egypt, Tanzania, Jordan, Poland, Ghana, and Iran (Díaz et al., 2010; Robertson et al., 2010; Yang et al., 2011; Rieux et al., 2013; Mahfouz et al., 2014; Tzanidakis et al., 2014; Parsons et al., 2015; Hijjawi et al., 2016; Mirhashemi et al., 2016; Kaupke et al., 2017; Squire et al., 2017; Majeed et al., 2018; Firoozi et al., 2019). In addition, the pertinent literatures about *C. ubiquitum* infection in sheep were derived from Ireland, Kuwait, Australia, Spain, Greece, Poland, Ghana, Iran, and Algeria (Díaz et al., 2010; Yang et al., 2011; Tzanidakis et al., 2014; Mirhashemi et al., 2016; Kaupke et al., 2017; Squire et al., 2017; Baroudi et al., 2018; Majeed et al., 2018; Firoozi et al., 2019). In China, *C. xiaoi* and *C. ubiquitum* were also found in sheep in Anhui, Xinjiang, Jilin, Inner Mongolia, Ningxia, Shandong, Shanghai, Henan, Qinghai, and Beijing (Mi et al., 2018; Qi et al., 2019), Tibetan sheep in Qinghai (Li et al., 2016), and goat in Guangdong, Hubei, Shandong, Shanghai, Henan, Chongqing, Shaanxi (Mi et al., 2014; Wang et al., 2014; Peng et al., 2016). Interestingly, *C. xiaoi* and *C. ubiquitum* have also been occasionally found in yak (Ma et al., 2014). The infection of *C. xiaoi* and *C. ubiquitum* in hosts is usually asymptomatic. However, the infection occasionally causes diarrhea and weight loss (Santín, 2013). More importantly, *C. xiaoi* is also found in HIV/AIDS patients (Adamu et al., 2014), and *C. ubiquitum*, previously known as the cervine genotype, has been emerging as another major zoonotic species that infects persons (Li et al., 2014), thus posing a risk to public health. Therefore, *C. xiaoi* and *C. ubiquitum* are of public health concern because of its wide geographic distribution and broad host range.

China has abundant biodiversity resources. Tibetan Antelope (*Pantholops hodgsonii*) is one of the most important wild animal species, which is a very important part of the natural ecology in Qinghai-tibet plateau (Peng et al., 2018). In 1981, China had accessed to the convention on international trade about endangered species of wild fauna and flora, in which the Tibetan antelope was classified into appendix I species (http://www.iucnredlist.org/). Since 1988, the Tibetan Antelope was identified as a first-grade state protection of wildlife (http://www.forestry.gov.cn/main/3954/content-1063883.html). However, the information for this pathogen infection in Tibetan Antelope is limited. Importantly, there has been no available information concerning *Cryptosporidium* infection in Tibetan Antelope. Therefore, the objective of the present study was to molecularly determine the prevalence and characterization of *Cryptosporidium* species in Tibetan Antelope in Tibet Autonomous Region, China.

## Materials and Methods

### Specimen Collection

**Figure 1:**
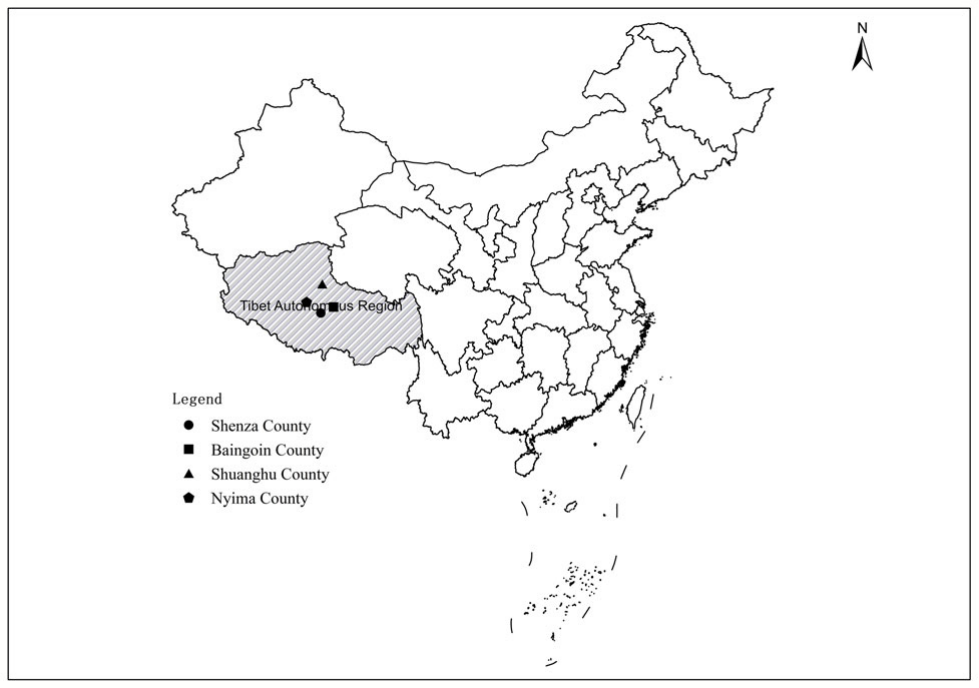
A map of Tibet Autonomous Region, China showing the geographical regions in Nyima County, Shuanghu County, Shenza County, and Baingoin County, in which Tibetan Antelope were sampled.

A total of 627 fecal samples of Tibetan Antelope were collected from Nyima County, Shuanghu County, Shenza County, and Baingoin County in Tibet Autonomous Region of China in 2019 and 2020 (**Figure 1**). A fresh fecal sample (approximately 5 g) for each Tibetan Antelope was collected from the ground using sterile gloves after defecation, and then was placed into ice boxes and sent to the laboratory. Tibetan Antelope with horns are males, otherwise, are females. The information regarding sampling time, region, and gender were recorded. This study was approved by the Ethics Committee of Jilin University.

### DNA Extraction and PCR Amplification

The fecal samples were diluted with 0.9% normal saline and filtered through 100-mesh stainless steel sieve. The filtrate was centrifuged at 4000 rpm/min for 5 min to enrich *Cryptosporidium* eggs. Genomic DNA was extracted from approximately 200 mg of each stool specimen using the E.Z.N.A.^®^ Stool DNA Kit (Omega Biotek Inc., Norcross, GA, USA) according to the manufacturer’s instructions, and then were stored at -20°C prior to a PCR analysis. *Cryptosporidium* prevalence and their species/genotypes were identified by nested PCR amplification of the small subunit ribosomal RNA (SSU rRNA) gene, using the primers 18SiCF2 (5′-GACATATCATTCAAGTTTCTGACC-3′) and 18SiCR2 (5′-CTGAAGGAGTAAGGAACAACC-3′) that amplified a fragment of about 760 bp in length in the first round PCR and the primers 18SiCF1 (5′-CCTATCAGCTTTAGACGGTAGG-3′) and 18SiCR1 (5′-TCTAAGAATTTCACCTCTGACTG-3′) that amplified a fragment of about 590 bp in length in the second round PCR (Qin et al., 2014; Koehler et al., 2018). The positive and negative controls were included in each test. The second PCR products were observed using UV light after electrophoresis at a 1.5% (m/V) agarose gel containing ethidium bromide.

### Sequence and Phylogenetic Analyses

The positive PCR products were sent to Sangon Biotech Company (Shanghai, China) for sequencing. The PCR products were sequenced on both strands to guarantee the accuracy of the sequence. A new PCR product was subjected to sequencing when single nucleotide substitution, insertion, or deletion was found in the former sequencing. The alignment and analysis for the SSU rRNA nucleotide sequences and reference sequences were performed using the Clustal X 1.83 program and Basic Local Alignment Search Tool (BLAST) (https://blast.ncbi.nlm.nih.gov), in order to determine the species of *Cryptosporidium*. The phylogenetic trees were reconstructed by MEGA 5.0 software using a neighbor-joining (NJ) method with a Kimura 2-parameter model (1,000 replicates). The representative nucleotide sequences were disposed to GenBank with accession numbers MZ220364 and MZ220365.

### Statistical Analysis

To assess the possible risk factors (gender, region, and year) associated with an exposure to *Cryptosporidium* infection in Tibetan Antelope, a multivariable logistic regression analysis was carried out using the PASW Statistics 18.0 (SPSS, Inc., IBM Corporation, Somers, NY) (Zhao et al., 2013). When independent variables were contained in the multivariable logistic regression model, probability (*P*) value < 0.05 was considered as statistically significant between levels within factors and interactions, and their odd ratio (OR) and 95% confidence interval (CI) were calculated.

## Results

### Prevalence and Risk Factors of *Cryptosporidium*

In the present study, 19 (3.0%, 95% CI 1.7–4.4) out of 627 Tibetan Antelope fecal samples from Tibet Autonomous Region were tested as *Cryptosporidium*-positive by PCR amplification of the SSU rRNA gene. The prevalence of *Cryptosporidium* infection in Tibetan Antelope was 2.2% (7/322, 95% CI 0.6–3.8) in 2019, and 3.9% (12/305, 95% CI 0.6–3.8) in 2020 (**Table 1**). Male Tibetan Antelope had a higher prevalence (3.7%, 95% CI 1.7–5.7, 12/327) as compared to that of females (2.3%, 95% CI 0.6–4.0, 7/300) (**Table 1**). The prevalence of *Cryptosporidium* in Tibetan Antelope in Nyima County, Shenza County, Shuanghu County, and Baingoin County was 3.8% (7/182, 95% CI 1.1–6.6), 4.8% (10/209, 95% CI 1.9–7.7), 0.9% (1/103, 95% CI 0.0–2.9), and 0.8% (1/133, 95% CI 0.0–2.2), respectively (**Table 1**).

**Table 1:** Prevalence and subtypes of *Cryptosporidium* infection in Tibetan Antelope (*Pantholops hodgsonii*) among different related factors.

Factor	Category	No. tested	No. positive	Prevalence(%) (95% CI)	*P* value	OR (95% CI)	Species/genotypes (no.)
Gender	Female	300	7	2.3 (0.6–4.0)	0.333	Reference	*Cryptosporidium ubiquitum* (2); *Cryptosporidium xiaoi* (5)
	Male	327	12	3.7 (1.7–5.7)		1.60 (0.62–4.11)	*Cryptosporidium ubiquitum* (10); *Cryptosporidium xiaoi* (2)
Sampling year	2019	322	7	2.2 (0.6–3.8)	0.205	Reference	*Cryptosporidium xiaoi* (7)
	2020	305	12	3.9 (1.8–6.1)		1.84 (0.72–4.75)	*Cryptosporidium ubiquitum* (12)
Region	Nyima County	182	7	3.8 (1.1–6.6)	0.160	5.28 (0.64–43.44)	*Cryptosporidium ubiquitum* (1); *Cryptosporidium xiaoi* (6)
	Shuanghu County	209	10	4.8 (1.9–7.7)		6.63 (0.84–52.43)	*Cryptosporidium ubiquitum* (10)
	Shenza County	103	1	0.9 (0.0–2.9)		1.29 (0.08–20.94)	*Cryptosporidium ubiquitum* (1)
	Baingoin County	133	1	0.8 (0.0–2.2)		Reference	*Cryptosporidium xiaoi* (1)
Total		627	19	3.0 (1.7–4.4)			*Cryptosporidium ubiquitum* (12); *Cryptosporidium xiaoi* (7)

According to multivariable logistic regression, gender, sampling year, and region of Tibetan Antelope were not significant in the logistic regression analysis (*P* > 0.05) and left out of the final model (Hosmer and Lemeshow goodness of fit test *P* = 1.00). Therefore, gender, sampling year, and region of collecting samples were not considered as main risk factor to influence the seroprevalence significantly (**Table 1**).

### Distribution and Phylogenetic Analysis of *Cryptosporidium*

**Figure 2:**
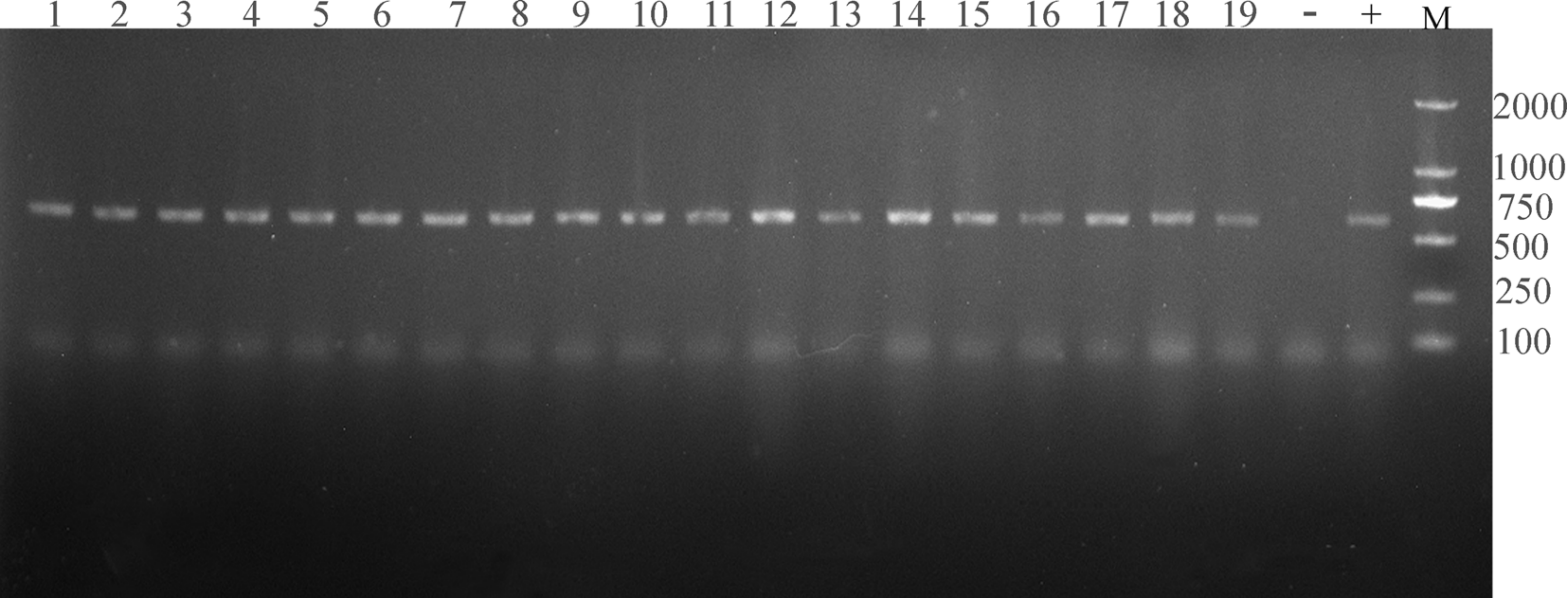
The electropherogram of PCR amplification of SSU rRNA gene of *Cryptosporidium*. Lanes 1–19 represent TA3, TA9, TA12, TA33, TA61, TA94, TA99, TA103, TA203, TA215, TA217, TA220, TA224, TA230, TA231, TA236, TA241, TA258, and TA301, respectively (19 *Cryptosporidium*-positive samples); “-” represents negative control; “+” represents positive control; “M” represents DL2000 DNA marker.

**Figure 3:**
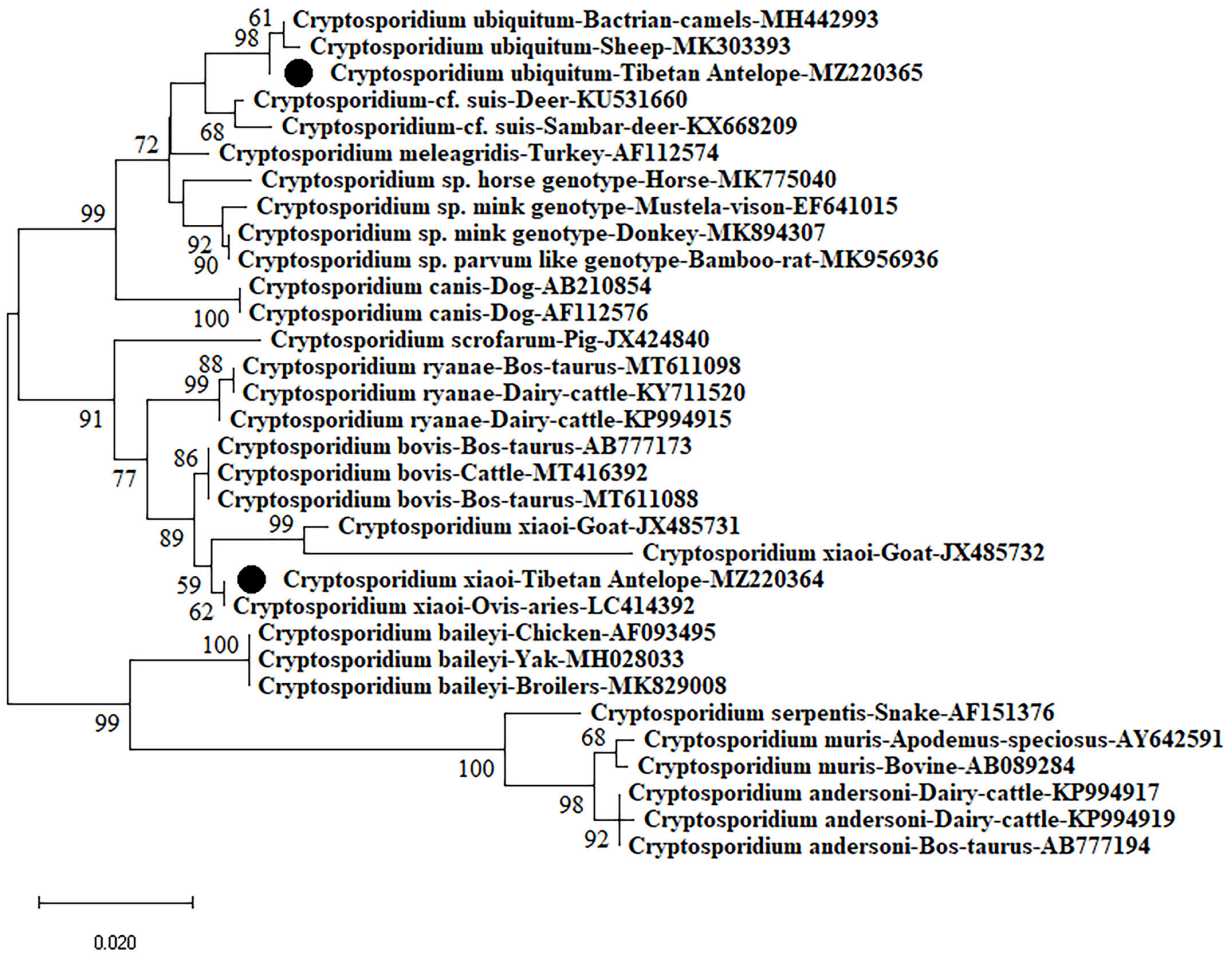
Phylogenetic analyses of *Cryptosporidium* using neighbor-joining (NJ) method (Kimura 2-parameter model). Bootstrap values below 50% are not shown (1,000 replicates). *Cryptosporidium* isolates identified in the present study are indicated by solid circles.

In the present study, 19 samples were *Cryptosporidium*-positive tested based on the SSU rRNA gene (**Figure 2**). The analysis of SSU rRNA gene suggested that the samples were *C. xiaoi* (n = 7) and *C. ubiquitum* (n = 12) positive in investigated Tibetan Antelope (**Table 1** and **Figure 3**). *C. ubiquitum* is the predominant *Cryptosporidium* species, which was responsible for 63.2%. *C. xiaoi* was only found in Nyima County (n = 6) and Baingoin County (n = 1) in 2019, and *C. ubiquitum* was only identified in three counties (n = 1 in Nyima County; n = 10 in Shuanghu County; n = 1 in Shenza County) in 2020 (**Table 1**). Moreover, *C. xiaoi* and *C. ubiquitum* were identified in both males (n = 12) and females (n = 7) in present study (**Table 1**). The representative sequences of *C. xiaoi* showed 100% similarity with sequences of *C. xiaoi* (MH049731, KF907825). The representative sequences of *C. ubiquitum* were identical to the sequences of *C. ubiquitum* (MT044147, MK573335).

## Discussion

The overall *Cryptosporidium* prevalence was 3.03%, which was significantly lower than that in sheep and goats in Kuwait (9.71%, 54/556) (Majeed et al., 2018), Jordan (10.53%, 12/114) (Hijjawi et al., 2016), Poland (24.78%, 84/339) (Kaupke et al., 2017), Spain (5.9%, 33/58) (Díaz et al., 2018), sheep in Iran (9.1%) (Haghi et al., 2020), goat in Australia (27.2%) (Al-Habsi et al., 2017), and Norwegian sheep in Norway (15%) (Robertson et al., 2010). It is also lower than that in sheep and/or goats in many provinces of China, such as goats in Henan and Chongqing (3.48%, 44/1256) (Wang et al., 2014), Guangdong, Hubei, Shandong, and Shanghai (11.4%, 69/604) (Mi et al., 2014), Tibetan sheep in Qinghai (12.3%, 43/350) (Li et al., 2016), sheep in 10 provinces of China (28.5%, 295/1035) (Mi et al., 2018), but higher than that of sheep in Xinjiang (0.9%, 3/318) (Qi et al., 2019). In the present study, statistical analysis showed that there was no significant difference in *Cryptosporidium* prevalence with several risk factors (*P* > 0.05), suggesting that gender, sampling year, and region may not be crucial factors for *Cryptosporidium* infection in Tibetan Antelope. The difference in *Cryptosporidium* prevalence may be related to sampling position, sensitivity of the employed detection method, sample sizes, susceptibility in different animals, the pollution degree of environment caused by *Cryptosporidium* oocysts, as well as animal husbandry practices.

*Cryptosporidium* genus consists of more than 108 species/genotypes. To date, *C. ryanae, C. bovis, C. xiaoi, C. parvum, C. andersoni, C. meleagridis, C. baileyi, C. hominis, C. ubiquitum, C. scrofarum, Cryptosporidium cervine* genotype, sheep genotype I, and *Cryptosporidium* rat genotype II have been reported in various sheep worldwide (Wang et al., 2010; Sweeny et al., 2011; Silverlås et al., 2012; Rieux et al., 2013; Koinari et al., 2014; Yang et al., 2014; Koinari et al., 2014; Mirhashemi et al., 2016; Kaupke et al., 2017; Sqquire et al., 2017; Firoozi et al., 2019). However, only *C. xiaoi* and *C. ubiquitum* were identified in Tibetan Antelope in this study, thus suggesting the *C. xiaoi* and *C. ubiquitum* were epidemic in the investigated Tibetan Antelope in Tibet Autonomous Region. Moreover, the sequences of isolates from seven fecal samples carrying *C. xiaoi* shared 100% similarity with isolates from sheep in the Algeria (LC414392) and China (MH049731), goats in Poland (KY055403), and Tibetan sheep in China (KF907825), showing that the sequences of *C. xiaoi* from Tibetan Antelope have a certain correlation with sheep and goat in Algeria, Poland, and China. But the detailed transmission chain of *Cryptosporidium* in Tibetan Antelope should be conducted in-depth study in the future. Similarly, another sequence of the 12 isolates belonging to *C. ubiquitum* showed 100% similarity with an isolate from cattle in the India (MT044147), goats in Algeria (LC414387), and Tibetan sheep in China (MK573335), indicating that the sequences of *C. ubiquitum* from Tibetan Antelope have a connection with cattle, goat, and sheep in India, Algeria, and China. More importantly, *C. ubiquitum* and *C. xiaoi* were also found in other animals and even in HIV/AIDS patients (Adamu et al., 2014; Li et al., 2014). According to relevant literature reports, *C. ubiquitum* was identified as six subtype families (XIIa–XIIf) based on the 60-kDa glycoprotein (gp60) gene (Li et al., 2014). Among them, subtype XIIa of *C. ubiquitum* was found in ruminants worldwide, subtype families XIIb–XIId of *C. ubiquitum* were found in rodents in the United States, and XIIe and XIIf of *C. ubiquitum* were found in rodents in the Slovak Republic (Li et al., 2014). In addition, humans were found to be infected with subtypes XIIa and XIIb–XIId isolates of *C. ubiquitum* (Li et al., 2014). In the investigated regions, the population of Tibetan Antelope lived with other free-range animals on the same prairie, and shared with the same source of water, which showing the risk of *Cryptosporidium* transmission between domestic and wild animals. Contacting with sheep infected with *C. ubiquitum* and drinking water contaminated by wildlife infected could be sources of human infections (Li et al., 2014). These findings not only demonstrated that *Cryptosporidium* infection of Tibetan Antelope may result from nearby animals, local herdsmen, or polluted water source, but also suggested that the Tibetan Antelope might be one of the important resources transmitting *Cryptosporidium* to local people and other native animals, including goa, blue sheep, yak, takin, and wapiti.

In addition, the Tibetan Antelope freely lived in high altitude regions, and frequently moved in plenty of space. They can also contact with other animals. Moreover, the shedding of oocysts into environment by Tibetan Antelope becomes the most important resource for a transmission to other animals and humans. As is well-known, *Cryptosporidium* is widely regarded as the pathogen of livestock, poultry, companion animals, and wildlife, posing a threat to public health. Local Tibetan live a herding life for chronically, which result in contacting with wildlife and free-range livestock frequently. Local Tibetan occasionally drink water in the process of grazing. Drinking untreated water contaminated by wildlife might be a potential source of *Cryptosporidium* infecting local Tibetan in Tibet Autonomous Region. Thus, it is very important to take actions for protecting Tibetan Antelope, other free-range animals, and local Tibetan from infecting with *Cryptosporidium* and the infection status of pathogens (not only *Cryptosporidium*) in Tibetan Antelope should continue to be monitored in the future. Further studies will sample more Tibetan Antelope in different regions to determine the dynamics and full profiles of *Cryptosporidium* infection in Tibetan Antelope, to examine the infection status of the local Tibetans with *Cryptosporidium*, and to assess the zoonotic potential of *Cryptosporidium* from Tibetan Antelope.

## Conclusions

This is the first report of *C. xiaoi* and *C. ubiquitum* infection in Tibetan Antelope worldwide. The overall prevalence of *Cryptosporidium* was 3.03%. The results also confirmed that *C. xiaoi* and *C. ubiquitum* were the most common *Cryptosporidium* species in Tibetan Antelope. Furthermore, *C. xiaoi* and *C. ubiquitum*, occasionally found in humans, were also identified in the Tibetan Antelope in this study. These results suggest the transmission of *Cryptosporidium* from Tibetan Antelope to other animals and/or humans should cause enough attention.

## Data Availability Statement

The datasets presented in this study can be found in online repositories. The names of the repository/repositories and accession number(s) can be found in the article/supplementary material.

## Ethics Statement

This study was approved by the Ethics Committee of Jilin University.

## Author Contributions

QZ, Y-GL, and W-QH conceived and designed the study and critically revised the manuscript. S-YQ, H-TS, J-HZ, Z-JW, and TM collected the samples. S-YQ, H-TS, and CL performed the experiments, analyzed the data, and drafted the manuscript. All authors read and approved the final manuscript.

## Funding

This work was supported by the “Independent research and development project from General Station of Forest and Grassland pest Management, National Forestry and Grassland Administration” (Grant No. LC-3-03), “Special Fund for Forestry Scientific Research in the Public Interest” (Grant No. 201504310), and “National Key Research and Development Program of China” (Grant no. 2017YFD0501706).

## Conflict of Interest

Author CL is employed by Shandong New Hope Liuhe Group Co., Ltd., and Qingdao Jiazhi Biotechnology Co., Ltd.

The remaining authors declare that the research was conducted in the absence of any commercial or financial relationships that could be construed as a potential conflict of interest.

## Publisher’s Note

All claims expressed in this article are solely those of the authors and do not necessarily represent those of their affiliated organizations, or those of the publisher, the editors and the reviewers. Any product that may be evaluated in this article, or claim that may be made by its manufacturer, is not guaranteed or endorsed by the publisher.
